# Routine CBC–based machine learning for individualized correction of lipemia-induced hemoglobin bias

**DOI:** 10.3389/fmed.2026.1975168

**Published:** 2026-10-29

**Authors:** Jiling Zhang, Xinjun Zhang, Yandan Wu

**Affiliations:** Department of Laboratory Medicine, Beijing Shunyi District Hospital, Shunyi Teaching Hospital of Capital Medical University, Beijing, China

**Keywords:** analytical interference, complete blood count, hemoglobin, lipemia, machine learning, random forest

## Abstract

**Background:**

Lipemia can interfere with photometric hemoglobin (HGB) measurement, causing spurious elevation and distortion of HGB-derived erythrocyte indices. We investigated whether routine complete blood count (CBC) parameters could continuously quantify lipemia-induced HGB bias and support individualized correction.

**Methods:**

We screened 310 clinical lipemic specimens from 310 unique patients measured on a Sysmex XN-9000. Twenty-seven specimens with a pre- versus post-plasma-replacement red blood cell (RBC) difference of ≥3% failed the prespecified procedural quality-control criterion and were excluded, leaving 283 specimens from 283 patients for analysis. Plasma-replacement HGB (HGB-PR) was used as the practical reference comparator. Relative bias was defined as Bias_PR = (HGB-I−HGB-PR)/HGB-PR × 100%. A random forest model used routine RBC count (RBC-I), routine photometric HGB (HGB-I), hematocrit (HCT-I), mean corpuscular hemoglobin (MCH-I), and mean corpuscular hemoglobin concentration (MCHC-I), with nested cross-validation (5 outer and 3 inner folds). Hyperparameters minimized the mean absolute error (MAE) of machine-learning-corrected HGB (HGB_ML) versus HGB-PR. All primary estimates used out-of-fold (OOF) predictions. Benchmarking, OOF SHapley Additive exPlanations (SHAP), lipid augmentation, and coupling sensitivity analyses were performed.

**Results:**

In the 283-specimen analysis cohort, the model achieved an OOF coefficient of determination (R^2^) of 0.848 for Bias_PR prediction (MAE, 1.20 percentage points; root mean square error [RMSE], 2.06). HGB_ML agreed strongly with HGB-PR (R^2^ = 0.975; MAE=1.66 g/L; RMSE=2.70 g/L; Lin concordance correlation coefficient [CCC] = 0.987), with 94.3% of errors ≤5 g/L. These favorable overall measures were not uniform across the interference range: underprediction and larger errors occurred in the high-Bias_PR tail. The random forest outperformed MCHC-only, ordinary least-squares, and Ridge models. MCHC-I dominated OOF SHAP importance (78.5%). Adding triglycerides (TG) and total cholesterol (TC) reduced HGB MAE to 1.48 g/L (*Δ*MAE, −0.177 g/L; 95% confidence interval [CI], −0.318 to −0.029). Strict anti-coupling retained useful average accuracy (MAE=1.71 g/L) but was less accurate at extreme interference. Formula-corrected HGB had lower analytical error than HGB_ML.

**Conclusions:**

Routine CBC parameters can support continuous estimation of lipemia-associated HGB bias and may provide an automated decision-support or reflex-testing signal. The model should not replace established analytical correction procedures, and independent multicenter prospective validation is required before clinical implementation.

## 1 Introduction

Lipemia is a common endogenous interference in clinical laboratory testing and can compromise the reliability of photometric measurements. In automated complete blood count (CBC) analysis, turbidity caused by circulating lipoprotein particles may spuriously increase measured hemoglobin (HGB), with secondary increases in calculated erythrocyte indices such as mean corpuscular hemoglobin (MCH) and mean corpuscular hemoglobin concentration (MCHC) (1–4). Importantly, the magnitude of optical interference is not determined by triglyceride (TG) concentration alone, because turbidity also depends on the concentration, size, and composition of lipoprotein particles; consequently, biochemical TG concentration and the actual degree of turbidity-related HGB interference can be discordant (1, 5, 6).

In routine laboratory practice, an abnormally increased MCHC is frequently used as an initial warning signal for lipemia or other analytical disturbances (2, 7, 8). However, increased MCHC is not specific for lipemia and may also occur with cold agglutination, hemolysis, abnormal erythrocyte morphology, and other analytical or biological conditions (3, 7, 8). Thus, a single MCHC threshold may flag suspicious results but cannot reliably quantify the magnitude of lipemia-associated HGB bias in an individual specimen.

For specimens with suspected lipemic interference, plasma replacement and plasma-HGB formula correction can provide more reliable HGB results (9–11). These approaches reduce analytical error but may require centrifugation, additional plasma measurement, or manual manipulation. A method that estimates the degree of HGB interference directly from information already generated by the routine CBC could therefore offer practical value as an automated decision-support layer.

Recent work illustrates the shift toward automated and data-driven management of lipemic CBC. Fan et al. developed an automated lipid-interference flagging strategy in clinical lipemic specimens (12). In our previous Sysmex XN-series study, Delta-HGB and MCHC-I effectively identified more severe lipemic interference (13). Li et al. subsequently used a random forest classifier to identify lipemic interference on the Mindray BC-6800 and proposed an HCT-based HGB correction equation (14). These studies establish the feasibility of automated recognition and correction, but binary classification does not quantify how much an individual specimen is biased, and a single regression equation may not capture nonlinear distortion across interrelated CBC parameters.

Accordingly, we hypothesized that routine CBC parameters could be used not only to flag lipemic interference but also to continuously estimate its magnitude. After application of the prespecified plasma-replacement RBC quality-control criterion, 283 specimens from 283 unique patients were analyzed using plasma-replacement HGB as a practical reference comparator. We developed and internally validated a CBC-only random forest model to predict continuous relative HGB bias and derive individualized machine-learning-corrected HGB. We compared this approach with simpler CBC models and conventional HGB correction strategies, examined its predictive structure using SHAP, and evaluated the incremental value of lipid measurements and the potential influence of mathematical coupling. Model development and reporting were guided by contemporary recommendations emphasizing separation of model optimization from performance evaluation, transparent uncertainty assessment, and cautious interpretation of machine-learning models (15–17).

## 2 Materials and methods

### 2.1 Study design and specimen cohort

This model-development and internal-validation study retrospectively screened clinical lipemic specimens collected at Beijing Shunyi District Hospital, Shunyi Teaching Hospital of Capital Medical University, between April 2025 and February 2026. A total of 310 lipemic specimens from 310 unique patients were initially screened on the Sysmex XN-9000 hematology platform. Each specimen represented an independent patient, and no repeated specimens or multiple measurements from the same patient were included in the analysis. Visible turbidity was independently assessed by two trained laboratory personnel, with discrepancies resolved by consensus. Specimens with potential analytical or biological confounders affecting HGB measurement or erythrocyte indices, including hemolysis, icterus, cold agglutination, hemoglobinopathies, marked red blood cell (RBC) morphological abnormalities, or other RBC disorders, were excluded. Twenty-seven specimens failed the prespecified plasma-replacement RBC quality-control criterion and were excluded, leaving 283 specimens from 283 unique patients in the final analysis cohort. All included specimens had complete routine CBC results, technically acceptable plasma-replacement measurements, formula-corrected HGB, and biochemical lipid measurements. The overall study workflow and exclusion process are shown in Figure 1, and definitions and units of the principal variables are provided in Supplementary Table S1.

**Figure 1:**
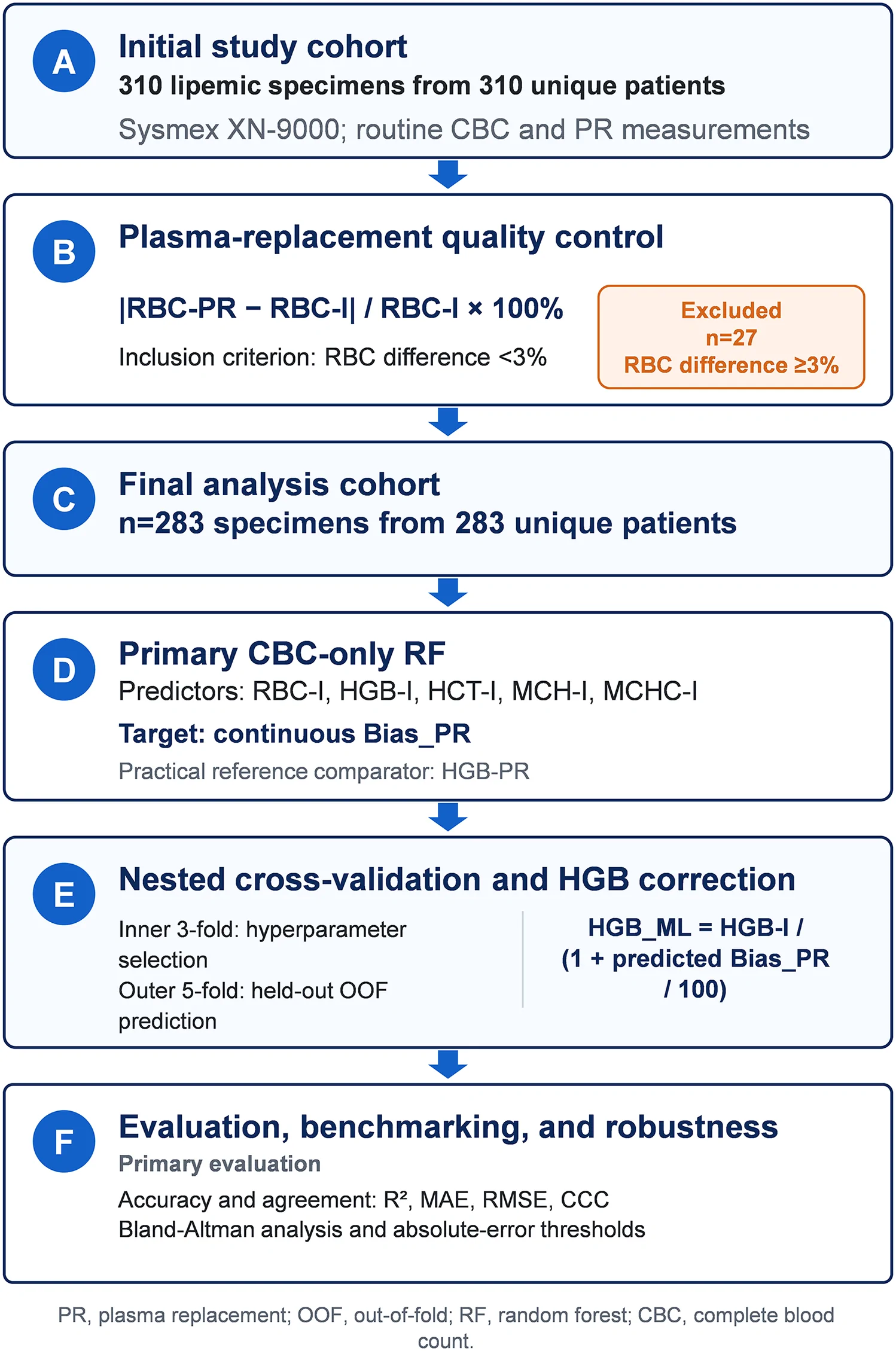
Study cohort, plasma-replacement quality control, model development, and evaluation workflow.

The primary objective was continuous estimation of lipemia-associated HGB bias and individualized HGB correction rather than binary detection of lipemia alone.

### 2.2 Hematologic measurements and plasma replacement

Routine hematologic measurements were obtained on the Sysmex XN-9000. The five CBC variables used in the primary model were RBC-I, HGB-I, HCT-I, MCH-I, and MCHC-I; HGB-I represented routine photometric HGB.

For plasma replacement, specimens were centrifuged at 1,500 × g for 10 min. Plasma was removed and replaced with an equal volume of diluent, followed by repeat analysis. Plasma-replacement HGB (HGB-PR) was treated as a practical reference comparator rather than an absolute gold standard. Replacement of the turbid plasma fraction directly reduces lipemia-related optical interference while preserving the cellular component, but the procedure remains susceptible to cell loss, volume mismatch, inadequate resuspension, and operator-dependent variation. Because RBC count should remain essentially unchanged when only the plasma fraction is replaced, a < 3% pre- versus post-replacement RBC difference was selected *a priori* as a conservative procedural consistency tolerance. Differences ≥3% were considered evidence of potentially important procedural or repeat-measurement variation. This cutoff was used solely to judge technical acceptability of plasma replacement; it was not a clinical decision threshold and was not optimized against model performance. Procedural acceptability was calculated as |RBC-PR−RBC-I|/RBC-I × 100%. Of the 310 candidate specimens, 27 had a difference ≥3% and were excluded before model development; 283 passed this criterion. HGB measured after technically acceptable plasma replacement was designated HGB-PR and used as the practical reference comparator for bias estimation. Formula-corrected HGB was additionally evaluated as an established analytical comparator.

Formula-corrected HGB was calculated according to the previously described approach (9):

HGB_formula=HGB_original−HGB_plasma×(1−HCT_original).

### 2.3 Definition of HGB bias and individualized corrected HGB

Relative routine-HGB bias was calculated with HGB-PR as the comparator:

Bias_PR = (HGB-I−HGB-PR)/HGB-PR × 100%.

A positive Bias_PR indicated overestimation of routine HGB relative to HGB-PR. Continuous Bias_PR was the primary modeling outcome. OOF-predicted Bias_PR was converted into an individualized machine-learning-corrected HGB estimate as follows:

HGB_ML = HGB-I/(1 + predicted Bias_PR/100).

For descriptive analyses, observed Bias_PR was categorized as ≤3%, >3%–5%, >5%–10%, or >10%. These categories were used only for descriptive and sensitivity analyses and were not used as model targets or predictors.

### 2.4 Data quality control and model development

All 283 included specimens had complete values for the primary predictors and outcome; no statistical imputation was required. The five primary CBC predictors were used without data-driven feature selection, and no scaling or normalization was applied before random-forest modeling. Before modeling, the distributions, Pearson correlations, and multicollinearity of the five CBC predictors were reviewed, including variance inflation factors. Because MCHC is mathematically related to HGB and HCT, reported MCHC-I was also compared with MCHC recalculated from HGB-I and HCT-I, and specimens with the largest discrepancies were reviewed. Quality-control findings are shown in Supplementary Figure S1.

Five-fold outer and three-fold inner nested cross-validation was used to reduce optimistic performance estimation associated with model tuning (17). Outer folds were approximately stratified across the Bias_PR distribution. Each forest contained 600 trees (18). Candidate maximum depths were 4, 6, 8, or unlimited; candidate min.bucket values were 2, 4, 6, 8, or 10, with min.node.size set to twice the corresponding min.bucket value; and candidate mtry proportions were 0.5, 0.7, or 1.0 of the available predictors. Eight combinations were sampled without replacement from this candidate grid within each inner cross-validation loop. Hyperparameters were selected by minimizing MAE between HGB_ML and HGB-PR rather than Bias_PR error alone.

After hyperparameter selection, a model was refitted within each complete outer training set and applied only to the corresponding outer test set. Thus, every specimen received one prediction from a model that had not been trained on that specimen.

### 2.5 Model performance and HGB correction evaluation

Continuous Bias_PR prediction was evaluated using the coefficient of determination (R^2^), mean absolute error (MAE), and root mean square error (RMSE), with Bias errors reported in percentage points. Scatter and residual plots were used to characterize prediction behavior across the interference range, and absolute Bias error was summarized across the four descriptive severity groups.

Agreement between HGB_ML and HGB-PR was evaluated using R^2^, MAE, RMSE, and Lin's concordance correlation coefficient (CCC) (19). Bland-Altman analysis was used to assess agreement and to estimate the mean difference and conventional 95% limits of agreement (20). The proportions of specimens with absolute HGB errors within ±3, ±5, ±7, and ±10 g/L were calculated to show the cumulative distribution of increasingly wide analytical error tolerances. For an HGB value near 120–150 g/L, these bounds represent approximate relative errors of 2%–3%, 3%–4%, 5%–6%, and 7%–8%, respectively. They were used for descriptive analytical interpretation only and should not be interpreted as validated clinical decision thresholds for anemia diagnosis, transfusion, or patient management. Ninety-five percent confidence intervals (CIs) for key performance measures were estimated using 2,000 patient-level bootstrap resamples; because each included specimen came from a unique patient, specimen- and patient-level resampling were equivalent.

### 2.6 Comparison with benchmark and conventional correction approaches

To assess the incremental value of nonlinear random-forest modeling, the primary model was compared with an MCHC-only linear model, a five-CBC ordinary least-squares model, and a five-CBC Ridge model (21). A simple HCT-only predictive model was also evaluated. All predictive comparators used the same outer-fold assignments as the primary model and generated OOF predictions.

The resulting HGB estimates were compared with uncorrected HGB-I and formula-corrected HGB using R^2^, MAE, RMSE, CCC, Bland-Altman measures, and absolute-error thresholds. Patient-level paired differences in absolute HGB error were summarized, with 95% confidence intervals estimated from 2,000 bootstrap resamples.

### 2.7 Model interpretation

SHapley Additive exPlanations (SHAP) was used to describe the predictive structure of the CBC random forest (22). SHAP analysis followed the outer-fold framework: SHAP values were calculated only for held-out outer-test specimens, using the corresponding outer-training feature matrix as the background data. Global feature importance was summarized by mean absolute SHAP value, and dependence plots were used to visualize associations between predictor values and their contributions to predicted Bias_PR. SHAP findings were interpreted only as model-level attribution patterns, not as evidence of biological mechanisms, independent effects, interactions, or causality. No formal pairwise SHAP interaction test was performed.

### 2.8 Lipid augmentation and mathematical-coupling sensitivity analyses

To determine whether biochemical lipid measurements provided additional information beyond routine CBC variables, log2-transformed TG and total cholesterol (TC) were added to the primary predictors to form a lipid-augmented random forest. The same outer test folds were used for evaluation.

Because HGB-I is present in the definition of Bias_PR and MCH and MCHC are mathematically related to HGB, mathematical-coupling sensitivity analyses were performed to examine the extent to which predictive performance might depend on these potentially coupled variables. With lipid measurements retained, a reduced-coupling model excluded HGB-I and MCH-I, whereas a strict anti-coupling model additionally excluded MCHC-I from Bias_PR prediction. These analyses were intended as sensitivity checks and were not interpreted as proving complete independence from algebraic relationships among HGB-derived variables.

The relationship between TG and observed Bias_PR was also evaluated using Spearman correlation, together with descriptive analyses of TG-Bias discordance. Biochemical TG strata were treated as descriptive categories and were not equated with analyzer hemolysis-icterus-lipemia (HIL) grades.

### 2.9 Statistical analysis

To characterize performance at the extremes, specimens were ranked by absolute HGB_ML error and the proportions with errors >5, >7, and >10 g/L were calculated. As a prespecified secondary analysis, Bias_PR >4% was treated as a binary endpoint. The 4% cutoff was chosen as a pragmatic marker of more-than-minor relative HGB bias: at HGB concentrations of approximately 120–150 g/L, a 4% relative bias corresponds to about 5–6 g/L, which is of the same order as the absolute-error summaries used in this study. The cutoff was not selected by optimizing the receiver operating characteristic (ROC) curve and is not proposed as a validated clinical decision limit. Continuous OOF-predicted Bias_PR was used as the ROC score, and sensitivity, specificity, positive predictive value, negative predictive value, and accuracy were calculated for the rule predicted Bias_PR >4%.

Continuous variables were summarized as median and interquartile range or mean and standard deviation, as appropriate, and categorical variables as counts and percentages. Pearson or Spearman correlation was used according to the analytical context. Statistical tests were two-sided. This study represents model development with internal validation only; nested cross-validation was not considered external validation, and no claims of external generalizability or prospective deployment were made (15, 16). Analyses were performed in R version 4.5.3. Random forest models were implemented using ranger version 0.18.0 (23), and OOF Kernel SHAP values were calculated using kernelshap version 0.9.1.

## 3 Results

### 3.1 Cohort characteristics and distribution of HGB bias

Of 310 candidate specimens from 310 unique patients, 27 (8.7%) failed the plasma-replacement RBC quality-control criterion and were excluded; 283 specimens from 283 patients comprised the final analysis cohort (Figure 1; Table 1). The cohort comprised 202 male (71.4%) and 81 female (28.6%) patients, with a median age of 45.0 years (IQR, 37.0–56.0). Median TG and TC concentrations were 8.17 mmol/L (IQR, 5.26–17.43) and 5.81 mmol/L (IQR, 5.08–7.18), respectively. Additional hematologic and reference measurements are summarized in Table 1.

**Table 1:** Characteristics of the 283 lipemic specimens from 283 unique patients included in the final model-development cohort after exclusion of 27 specimens that failed the prespecified plasma-replacement RBC quality-control criterion. Continuous variables are presented as median [interquartile range] unless otherwise indicated.

Characteristic	Overall (*n* = 283)
Sex, *n* (%)	
Male	202 (71.4%)
Female	81 (28.6%)
Age (years)	45.0 [37.0–56.0]
Triglycerides (mmol/L)	8.17 [5.26–17.43]
Total cholesterol (mmol/L)	5.81 [5.08–7.18]
RBC-I (10^12^/L)	4.84 [4.47–5.16]
HGB-I (g/L)	152.0 [140.5–164.0]
HCT-I (%)	43.4 [40.6–46.6]
MCH-I (pg)	31.2 [30.2–32.5]
MCHC-I (g/L)	344.0 [336.0–358.0]
HGB-PR (g/L)	146.0 [134.5–158.0]
Formula-corrected HGB (g/L)	147.0 [136.0–159.0]
Bias_PR (%)	1.53 [0.74–3.79]

Observed Bias_PR remained markedly right-skewed (Figure 2A). The median Bias_PR was 1.53%, the mean was 3.68%, and the maximum was approximately 43.5%, indicating that most included specimens had relatively small HGB bias whereas a smaller subgroup showed substantial interference.

**Figure 2:**
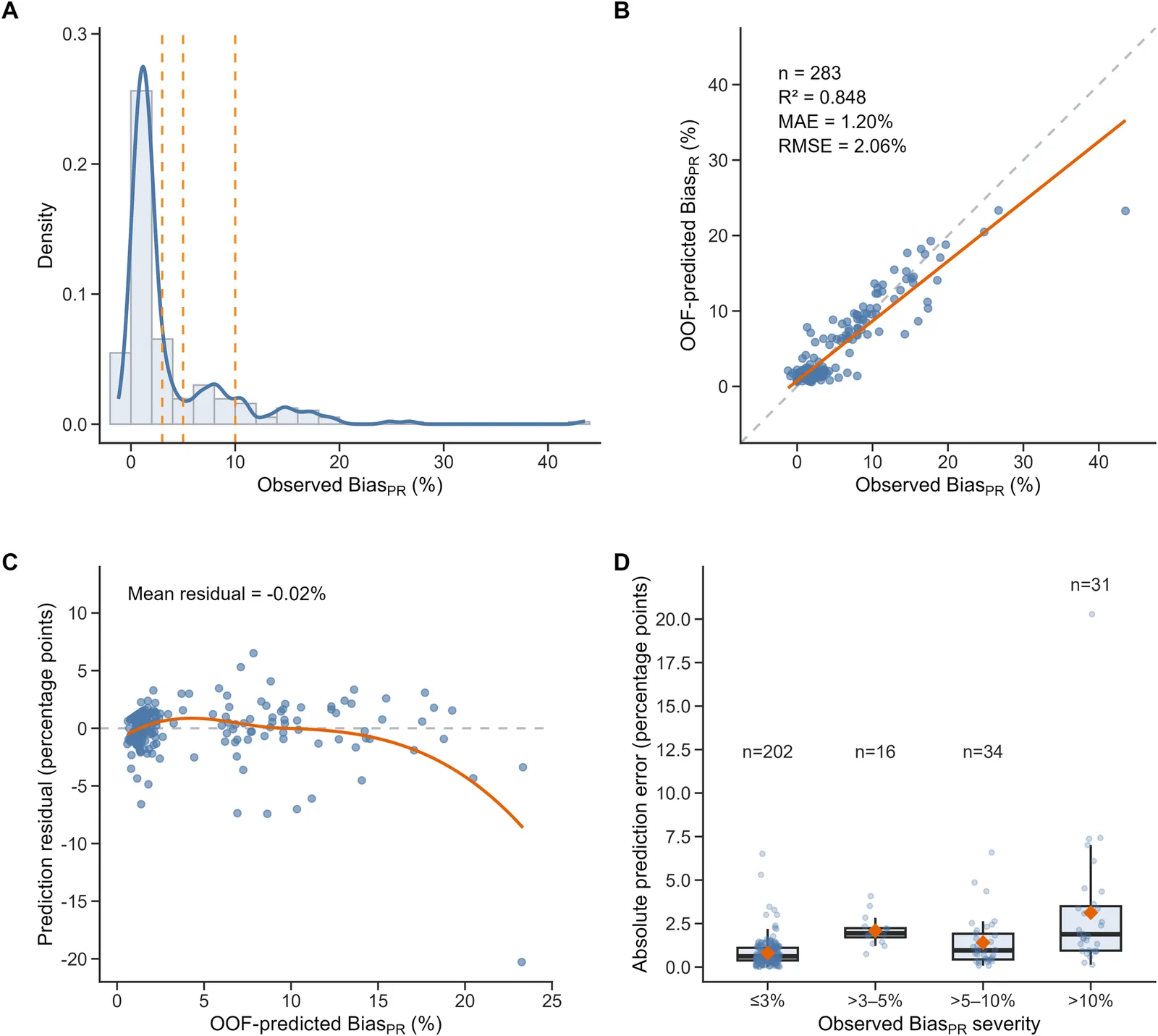
Out-of-fold prediction performance and error characteristics of the primary CBC-only random forest. **(A)** Distribution of observed Bias_PR; vertical reference lines denote descriptive severity thresholds of 3%, 5%, and 10%. **(B)** Observed versus OOF-predicted Bias_PR. The dashed diagonal line denotes perfect agreement and the fitted line summarizes the prediction trend. **(C)** Prediction residuals, defined as predicted minus observed Bias_PR; increasingly negative residuals at higher observed Bias_PR indicate progressive underprediction. **(D)** Absolute OOF prediction error across observed Bias_PR severity groups. Group sizes were *n* = 202, 16, 34, and 31 for the ≤3%, >3%–5%, >5%–10%, and >10% categories, respectively. All performance estimates were derived from nested-cross-validation OOF predictions.

### 3.2 CBC-based bias prediction and individualized HGB correction

Using the pooled OOF predictions from all 283 specimens, the CBC random forest achieved an R^2^ of 0.848 for continuous Bias_PR, with an MAE of 1.20 percentage points and RMSE of 2.06 percentage points (Figure 2B). Bootstrap analysis yielded a 95% CI of 0.797–0.904 for R^2^ and 1.04–1.41 percentage points for MAE. These overall estimates should be interpreted together with the residual and severity-stratified analyses, because performance deteriorated in the high-Bias_PR tail.

Although the overall mean prediction residual was close to zero (−0.02 percentage points), residuals became progressively more negative as OOF-predicted Bias_PR increased, indicating greater underprediction among specimens with higher predicted bias (Figure 2C). The 283 specimens were distributed across the four prespecified Bias_PR severity categories as follows: ≤3%, *n* = 202 (71.4%); >3%–5%, *n* = 16 (5.7%); >5%–10%, *n* = 34 (12.0%); and >10%, *n* = 31 (11.0%). Bias_PR MAE across these groups was 0.81, 2.08, 1.41, and 3.13 percentage points, respectively. The largest error was observed in the >10% group, and absolute prediction error increased with interference severity (Spearman rho=0.217, *P* < 0.001; Figure 2D).

After conversion of predicted Bias_PR to HGB_ML, overall agreement with HGB-PR was strong: R^2^ = 0.975, MAE=1.66 g/L, RMSE=2.70 g/L, and CCC=0.987 (Figure 3A). Bootstrap 95% CIs were 0.955–0.986 for R^2^, 1.45–1.91 g/L for MAE, 2.06–3.51 g/L for RMSE, and 0.977–0.993 for CCC. However, these global values did not imply uniform accuracy, because larger errors were concentrated at higher interference levels.

**Figure 3:**
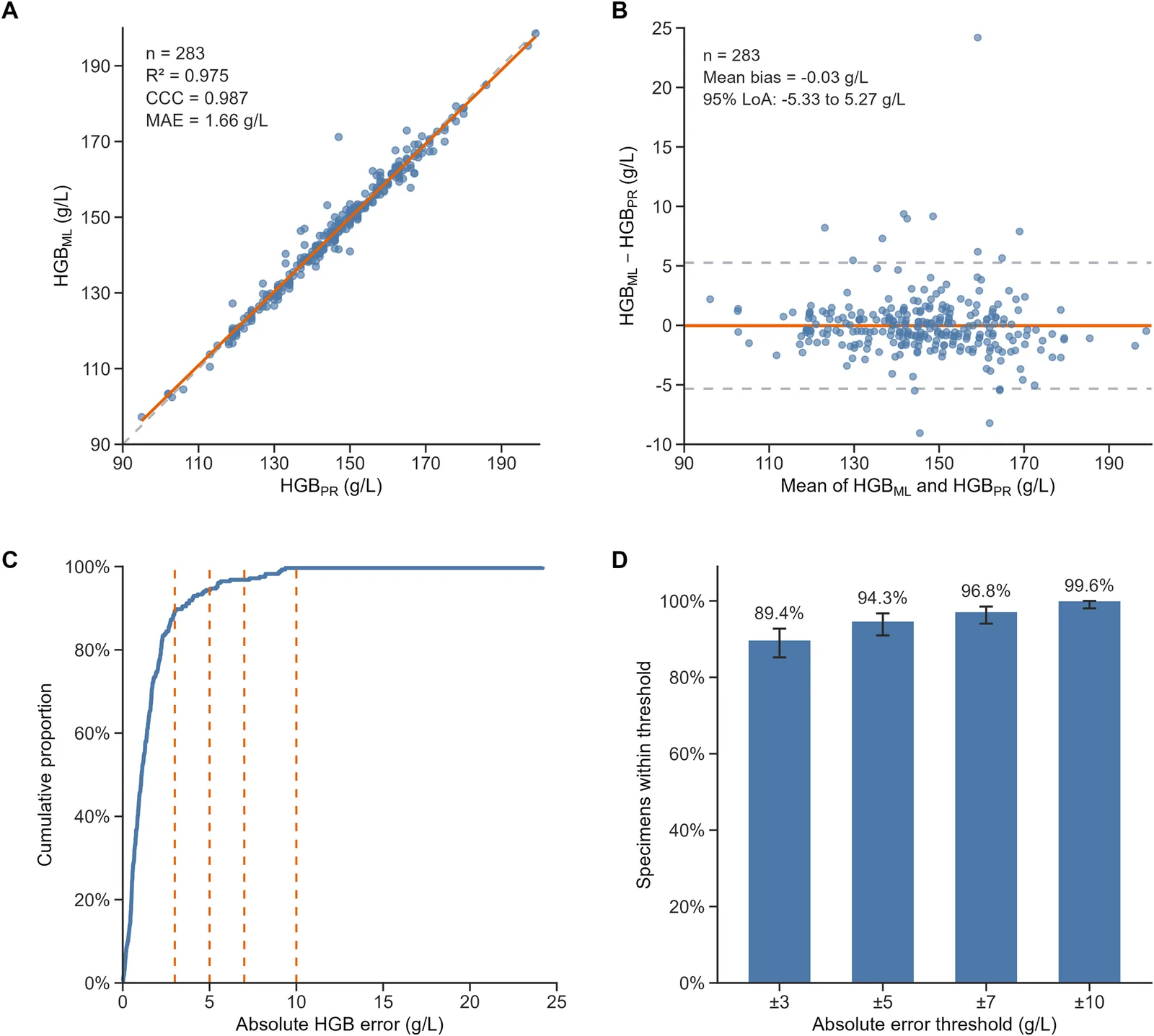
Accuracy and agreement of individualized machine-learning-corrected hemoglobin (HGB_ML). **(A)** OOF HGB_ML versus plasma-replacement HGB (HGB-PR), with the line of identity shown for reference. **(B)** Bland-Altman analysis of HGB_ML and HGB-PR; the solid line indicates mean difference and dashed lines indicate the 95% limits of agreement. **(C)** Empirical cumulative distribution of absolute HGB_ML error. **(D)** Proportions of specimens with absolute HGB_ML error within ±3, ±5, ±7, and ±10 g/L. These cutoffs summarize progressively wider analytical error tolerances and are not validated clinical decision limits. HGB-PR served as the practical reference comparator.

Bland-Altman analysis showed a mean HGB_ML−HGB-PR difference of −0.03 g/L with 95% limits of agreement from −5.33 to 5.27 g/L (Figure 3B). Absolute HGB error was ≤3, ≤5, ≤7, and ≤10 g/L in 89.4%, 94.3%, 96.8%, and 99.6% of specimens, respectively (Figures 3C,D).

### 3.3 Comparison with benchmark and conventional HGB correction approaches

The MCHC-only, CBC ordinary least-squares, and CBC Ridge models yielded HGB MAEs of 2.72, 2.64, and 2.63 g/L, respectively, compared with 1.66 g/L for the CBC random forest (Figure 4; Table 2). Corresponding Bias_PR R^2^ values were 0.761, 0.772, 0.775, and 0.848, respectively.

**Figure 4:**
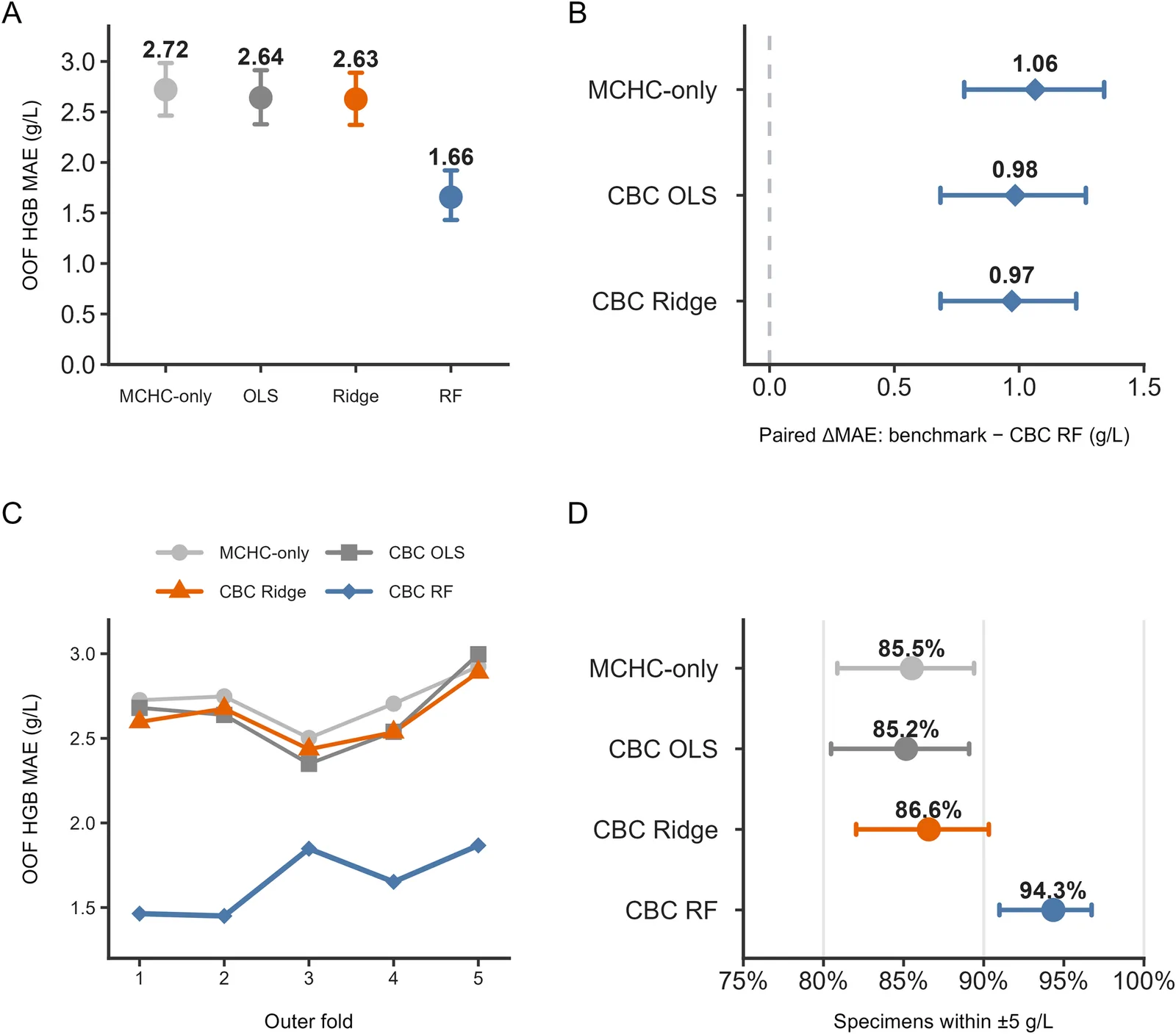
Benchmark comparison of the primary CBC-only random forest with simpler CBC-based models. **(A)** OOF HGB MAE with bootstrap 95% confidence intervals for the MCHC-only, CBC ordinary least-squares (OLS), CBC Ridge, and CBC random forest models. **(B)** Paired patient-level difference in absolute HGB error relative to the CBC random forest; positive values indicate lower MAE for the random forest. **(C)** Fold-specific HGB MAE across the five outer folds. **(D)** Proportion of specimens with absolute HGB error within 5 g/L. All benchmark models were evaluated in the same outer test folds.

**Table 2:** Same-fold out-of-fold predictive performance of the MCHC-only, CBC OLS, CBC ridge, and CBC RF models for continuous bias_PR and individualized HGB correction.

Model	Bias R^2^	Bias MAE (pp)	Bias RMSE (pp)	HGB R^2^	HGB MAE (g/L)	HGB RMSE (g/L)	CCC	Mean error (g/L)	Within ±5 g/L
MCHC-only	0.761	1.96	2.59	0.957	2.72	3.52	0.977	−0.16	85.5%
CBC OLS	0.772	1.89	2.53	0.957	2.64	3.50	0.978	−0.06	85.2%
CBC Ridge	0.775	1.88	2.51	0.959	2.63	3.44	0.979	−0.08	86.6%
CBC RF	**0**.**848**	**1**.**20**	**2**.**06**	**0**.**975**	**1**.**66**	**2**.**70**	**0**.**987**	**−0**.**03**	**94**.**3%**

Bold values indicate the performance estimates of the prespecified primary CBC-only random forest (CBC RF) model.

In paired patient-level analyses, the random forest reduced HGB MAE by 1.064 g/L relative to the MCHC-only model (95% CI, 0.780–1.341), 0.984 g/L relative to CBC ordinary least squares (95% CI, 0.685–1.267), and 0.971 g/L relative to CBC Ridge (95% CI, 0.685–1.229) (Figure 4B). The proportion within 5 g/L was 94.3% for the random forest, compared with 85.5%, 85.2%, and 86.6% for the three benchmark models (Figure 4D). The relative advantage of the random forest was broadly maintained across the five outer folds (Figure 4C; Supplementary Table S2).

Compared with routine and conventional HGB approaches (Table 3), uncorrected HGB-I had an MAE of 5.39 g/L, the HCT-only model 3.31 g/L, and the CBC random forest 1.66 g/L. Formula-corrected HGB had the lowest analytical error among the evaluated methods, with an MAE of 1.24 g/L. Thus, CBC-based machine-learning correction substantially reduced error relative to uncorrected HGB-I and outperformed the simple HCT-based prediction, but did not exceed formula correction in analytical accuracy.

**Table 3:** Comparison of uncorrected HGB-I, HCT-only OOF prediction, CBC-only RF HGB_ML, and formula-corrected HGB against HGB-PRin the 283-specimen cohort.

Method	R^2^	MAE, g/L (95% CI)	RMSE (g/L)	CCC	Mean bias (g/L)	95% LoA (g/L)	≤3 g/L	≤5 g/L	≤7 g/L	≤10 g/L
HGB-I uncorrected	0.697	5.39 (4.53–6.31)	9.32	0.871	5.36	−9.62 to 20.34	66.8%	73.5%	77.4%	80.2%
HCT-only OOF	0.935	3.31 (2.97–3.63)	4.33	0.966	0.00	−8.50 to 8.50	57.2%	78.8%	92.6%	97.5%
CBC-only RF HGB_ML	**0**.**975**	**1.66** (**1.44–1.91)**	**2**.**70**	**0**.**987**	**−0**.**03**	**−5.33 to 5.27**	**89**.**4%**	**94** **.** **3%**	**96**.**8%**	**99**.**6%**
Formula-corrected HGB	0.991	1.24 (1.12–1.36)	1.64	0.995	0.94	−1.70 to 3.58	97.9%	99.6%	100%	100%

Bold values indicate the performance estimates of the prespecified primary CBC-only random forest (CBC RF) model.

### 3.4 Model interpretation, lipid augmentation, and robustness analyses

OOF Kernel SHAP analysis assigned the largest mean absolute model-attribution value to MCHC-I (Figures 5A,B). Its mean absolute SHAP value was 2.98 percentage points, accounting for 78.5% of the total mean absolute SHAP magnitude across the five CBC variables, and MCHC-I ranked first overall. MCH-I ranked second (mean absolute SHAP, 0.36 percentage points), followed by HGB-I (0.20), HCT-I (0.17), and RBC-I (0.09). SHAP dependence plots described a pronounced nonlinear association between MCHC-I and its contribution to predicted Bias_PR (Figures 5C,D). These patterns characterize how the fitted model used correlated inputs and do not establish biological mechanisms, independent effects, or causality.

**Figure 5:**
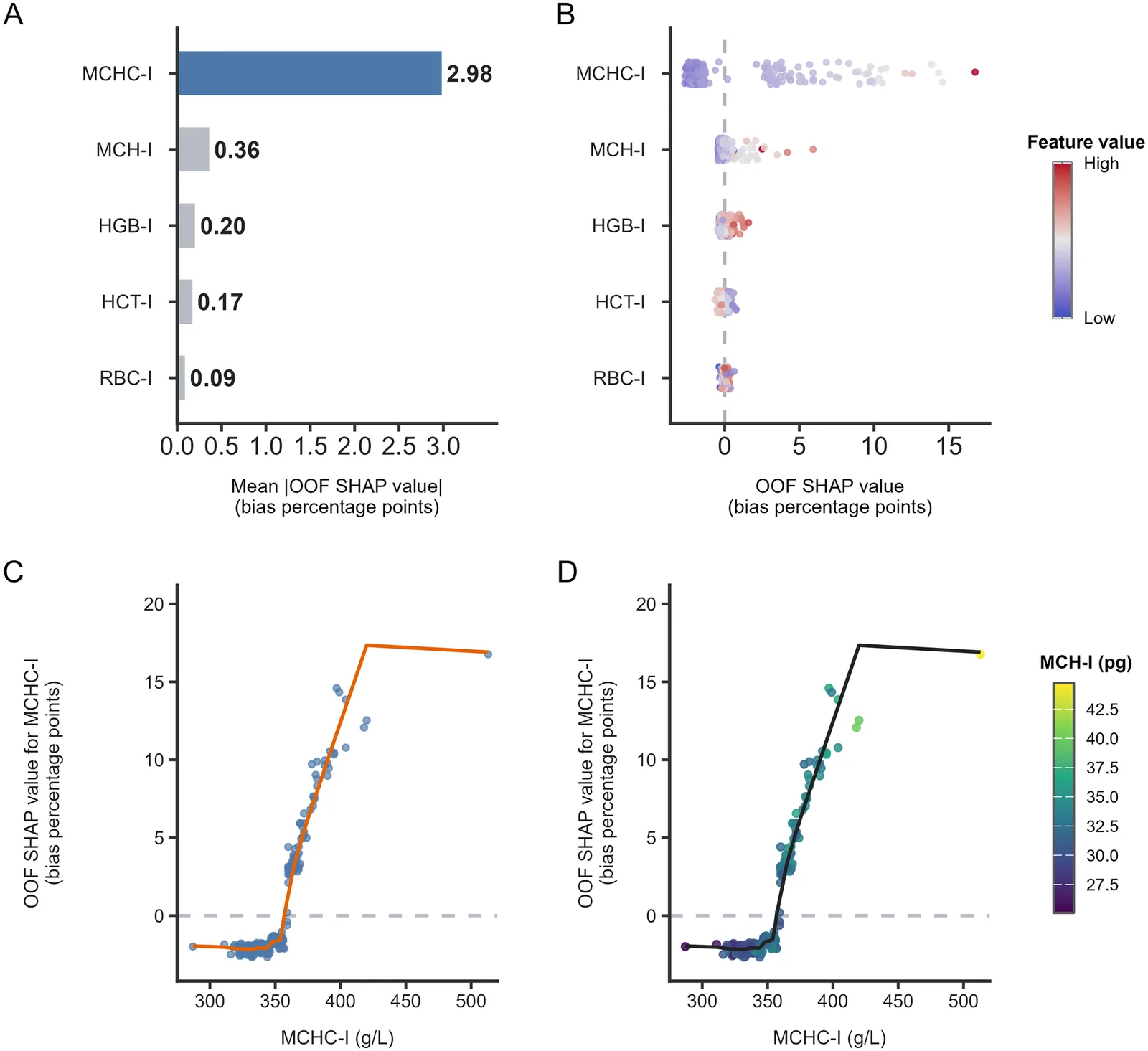
Out-of-fold kernel SHAP interpretation of the primary CBC-only random forest in the 283-specimen cohort. **(A)** Global feature importance summarized by pooled OOF mean absolute SHAP values; MCHC-I accounted for 78.5% of the total mean absolute SHAP magnitude. **(B)** OOF SHAP summary plot showing the direction and magnitude of feature contributions to predicted Bias_PR. **(C)** MCHC-I SHAP dependence plot demonstrating a pronounced nonlinear contribution pattern. **(D)** MCHC-I dependence plot colored by MCH-I for exploratory visualization of the broader erythrocyte-index context. No formal pairwise SHAP interaction test was performed, and SHAP patterns were interpreted as predictive structure rather than independent biological or causal effects.

Adding log2-transformed TG and TC provided a modest improvement (Figures 6A,B; Table 4). HGB MAE decreased from 1.66 to 1.48 g/L, HGB R^2^ increased from 0.975 to 0.978, and CCC increased from 0.987 to 0.989. The paired change in HGB MAE was −0.177 g/L (95% CI, −0.318 to −0.029). Severity-specific HGB MAE decreased from 1.16 to 1.09 g/L in the ≤3% group, from 3.03 to 2.06 g/L in the >3%–5% group, from 1.97 to 1.67 g/L in the >5%–10% group, and from 3.83 to 3.54 g/L in the >10% group (Figure 6D).

**Figure 6:**
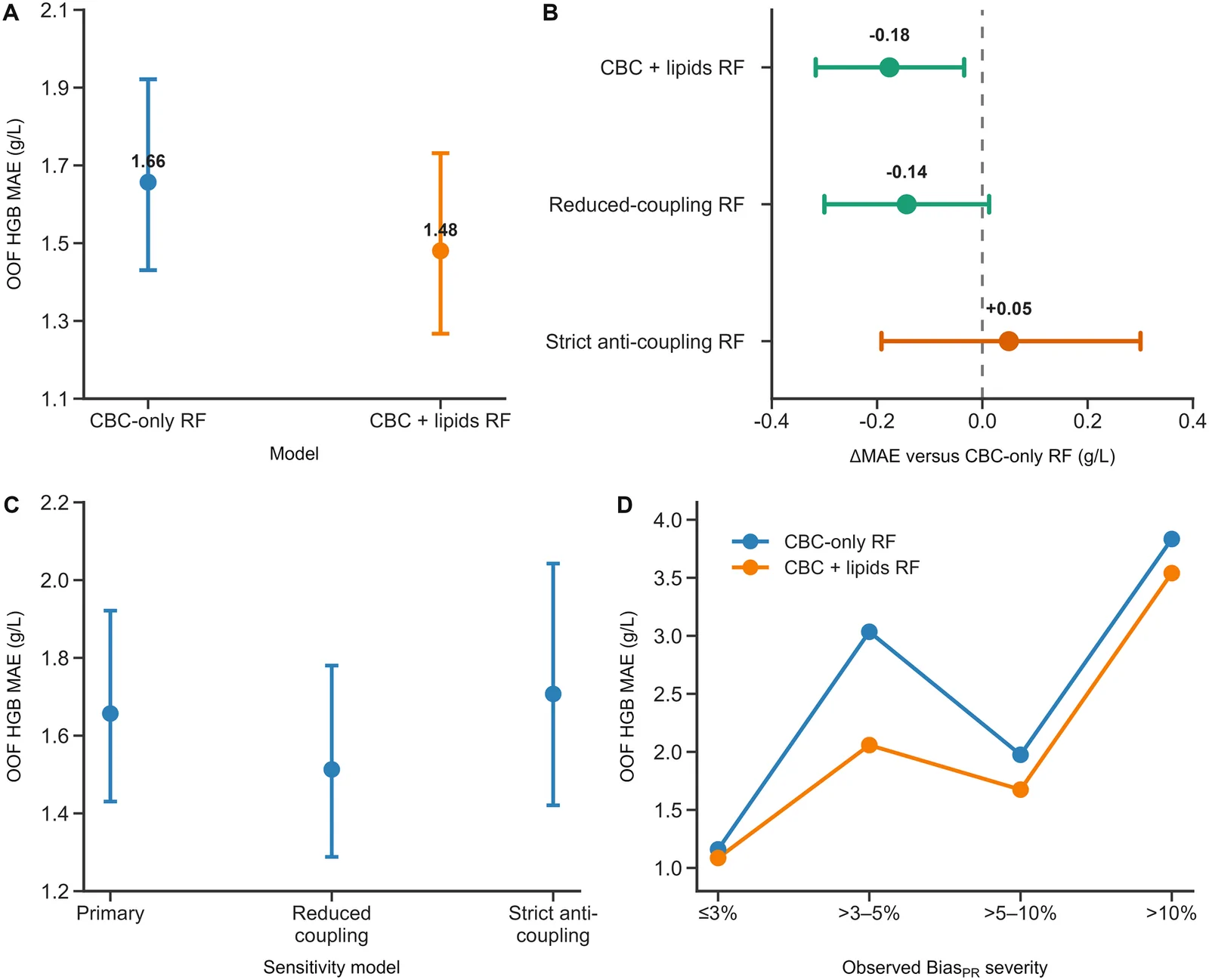
Incremental contribution of lipid measurements and robustness of the CBC-based prediction framework to mathematical coupling. **(A)** OOF HGB MAE with bootstrap 95% confidence intervals for the primary CBC-only random forest and the lipid-augmented model including log2-transformed triglycerides and total cholesterol. **(B)** Paired patient-level differences in absolute HGB error between each sensitivity model and the primary CBC-only random forest; negative values indicate lower MAE for the alternative model. **(C)** Comparison of HGB MAE for the primary, reduced-coupling, and strict anti-coupling models. The reduced-coupling model excluded HGB-I and MCH-I, whereas the strict anti-coupling model additionally excluded MCHC-I from Bias_PR prediction. **(D)** Severity-specific HGB MAE for the primary CBC-only and lipid-augmented models according to observed Bias_PR. All sensitivity models were evaluated in the same outer folds.

**Table 4:** Robustness and sensitivity analyses evaluating lipid augmentation and the influence of mathematical coupling on continuous bias_PR prediction and individualized HGB correction.

Model	Bias R^2^	Bias MAE (pp)	HGB R^2^	HGB MAE (g/L)	HGB RMSE (g/L)	CCC	Within ±5 g/L	*Δ*MAE vs. primary (g/L)	95% CI
CBC-only RF	**0**.**848**	**1**.**20**	**0**.**975**	**1**.**66**	**2**.**70**	**0**.**987**	**94**.**3%**	**Reference**	**—**
CBC+lipids RF	0.863	1.09	0.978	1.48	2.50	0.989	96.1%	−0.177	−0.318 to −0.029
Reduced-coupling RF	0.848	1.12	0.976	1.51	2.61	0.988	96.1%	−0.144	−0.294 to 0.017
Strict anti-coupling RF	0.770	1.28	0.964	1.71	3.20	0.982	92.9%	+0.050	−0.199 to 0.300

Bold values indicate the performance estimates of the prespecified primary CBC-only random forest (CBC RF) model.

In the mathematical-coupling sensitivity analyses, the reduced-coupling model excluding HGB-I and MCH-I achieved an HGB MAE of 1.51 g/L; its paired change versus the primary model was −0.144 g/L (95% CI, −0.294 to 0.017). The strict anti-coupling model, which additionally excluded MCHC-I from Bias_PR prediction, achieved an HGB MAE of 1.71 g/L; the paired change versus the primary model was +0.050 g/L (95% CI, −0.199 to 0.300) (Figures 6B,C; Table 4). However, the strict model's Bias_PR R^2^ decreased from 0.848 to 0.770 and HGB RMSE increased from 2.70 to 3.20 g/L. In specimens with Bias_PR >10%, HGB MAE increased from 3.83 to 5.04 g/L, indicating that directly HGB-related CBC indices contributed particularly to stable prediction at the extreme end of the interference distribution.

### 3.5 Supplementary and secondary analyses

The descriptive calibration intercept was −0.25 and the slope was 1.07. No major global calibration failure was evident, although the underprediction pattern at the highest Bias_PR values remained apparent.

TG was strongly but incompletely associated with observed Bias_PR (Spearman rho=0.734, *P* < 0.001; Supplementary Figure S2). Relative to a simple log2(TG)-only relationship, 12 specimens had observed Bias_PR more than 5 percentage points lower than expected and another 12 more than 5 percentage points higher, indicating that biochemical TG concentration alone did not fully represent the magnitude of HGB interference.

Performance in severe and extreme interference cases was examined separately. Among the 31 specimens with Bias_PR >10%, Bias_PR MAE was 3.13 percentage points, higher than in the lower-severity categories overall. In the full cohort, 16 specimens (5.7%) had an absolute HGB_ML error >5 g/L, 9 (3.2%) had an error >7 g/L, and 1 (0.4%) had an error >10 g/L; the maximum absolute error was 24.2 g/L (Supplementary Figure S3; Supplementary Table S2). The largest errors occurred disproportionately among specimens with greater observed Bias_PR, consistent with the progressive underprediction seen at the upper end of the interference distribution.

In the prespecified secondary classification analysis, 70 of 283 specimens (24.7%) had Bias_PR >4%. OOF-predicted Bias_PR achieved an area under the ROC curve (AUC) of 0.963, whereas MCHC-I alone achieved an AUC of 0.972 (Supplementary Figure S4). Using predicted Bias_PR >4% as the decision rule yielded a sensitivity of 91.4%, specificity of 97.7%, positive predictive value of 92.8%, negative predictive value of 97.2%, and accuracy of 96.1%. Because the 4% threshold was pragmatic rather than clinically validated and MCHC-I alone already provided strong binary discrimination, classification was retained as a secondary analysis rather than the principal modeling objective.

## 4 Discussion

In this study, we developed and internally validated a CBC-only framework for continuous quantification of lipemia-associated HGB bias and individualized HGB correction in 283 real-world clinical lipemic specimens that passed the prespecified plasma-replacement RBC quality-control criterion. Four findings are particularly important. First, routinely generated erythrocyte parameters contained sufficient information to estimate continuous Bias_PR with good OOF performance, and conversion of predicted bias into HGB_ML substantially reduced routine-HGB error. Second, the nonlinear random forest consistently outperformed MCHC-only and linear multivariable benchmarks. Third, MCHC-I dominated model attribution, but its contribution was nonlinear, indicating that the gain from the random forest could not be reduced to application of a single MCHC threshold. Fourth, lipid measurements provided only modest additional improvement, while useful predictive information persisted after removal of the CBC indices most directly coupled to HGB. Collectively, these findings support the concept that the multivariable pattern of distortion within the routine CBC contains information about the magnitude of lipemic HGB interference, not merely its presence.

Most established approaches to lipemia-associated HGB interference have focused on recognition of affected specimens or direct analytical correction. Plasma replacement and formula-based correction can reduce the influence of plasma turbidity on photometric HGB, but they require additional manipulation or measurements (9–11). Recent studies have increasingly explored automated strategies. Radišić Biljak et al. evaluated practical approaches to handling lipemic CBC samples on Sysmex analyzers (11). Fan et al. developed an automated interference-flagging strategy in clinical lipemic specimens (12). Our previous XN-series work showed that Delta-HGB and MCHC-I can identify more severe lipemic interference (13). Li et al. used random forest classification to identify interference and proposed an HCT-based HGB correction equation on the Mindray BC-6800 (14). The present study addresses a different question: rather than determining only whether interference is present, it treats interference magnitude as a continuous analytical phenotype and links predicted Bias_PR directly to a specimen-specific corrected HGB estimate. This distinction is reinforced by the secondary classification analysis, in which MCHC-I alone achieved an AUC of 0.972; if the sole objective were binary detection, a more complex model would provide limited additional value.

The dominance of MCHC-I in the model is analytically plausible. Lipemia primarily causes positive interference in photometric HGB, whereas HCT is not affected through the same optical mechanism; because MCHC reflects the relationship between HGB and HCT, disproportionate HGB elevation can manifest as an abnormal MCHC. This is why MCHC has long been used as an internal consistency check for suspicious erythrocyte results (1, 3, 7, 8). In the OOF SHAP analysis, MCHC-I accounted for 78.5% of total mean absolute SHAP magnitude. Nevertheless, the MCHC-only model produced an HGB MAE of 2.72 g/L, compared with 1.66 g/L for the full random forest. The multivariable benefit therefore appears to arise primarily from nonlinear modeling of the dominant MCHC-I signal while incorporating smaller contextual contributions from the remaining erythrocyte indices, rather than from evenly distributed importance across all five predictors.

These SHAP findings require cautious interpretation. RBC-I, HGB-I, HCT-I, MCH-I, and MCHC-I are correlated and, in several instances, mathematically derived from one another. A large SHAP value for MCHC-I should therefore not be interpreted as evidence that MCHC has an independent biological or causal role in lipemic interference. A more appropriate interpretation is that MCHC-I functions as an informative summary marker of disrupted internal consistency among erythrocyte measurements. Likewise, variation in the MCHC-I dependence pattern across MCH-I values represents model-level predictive context rather than a formally established biological interaction. No formal pairwise SHAP interaction test was performed, and we intentionally avoid claims regarding a 'strongest' SHAP interaction.

From a clinical laboratory perspective, the machine-learning correction should not be positioned as analytically superior to established direct correction methods. HGB_ML reduced MAE from 5.39 g/L for uncorrected HGB-I to 1.66 g/L and outperformed the HCT-only prediction, but formula correction retained a lower MAE of 1.24 g/L. Therefore, when formula correction can be implemented reliably, it remains the more accurate analytical solution. The potential value of the CBC-only approach lies elsewhere: it requires no additional specimen manipulation or lipid measurement and can generate an immediate quantitative estimate of likely HGB bias from data that are already available. After independent validation, such a model may be most useful as an automated decision-support layer for identifying specimens that warrant confirmation, reflex testing, or direct correction rather than as a replacement for established analytical procedures. Importantly, the present study evaluated analytical performance rather than direct clinical utility. Agreement with HGB-PR, prediction error, and related analytical metrics do not establish that model use improves anemia classification, transfusion decisions, patient management, or clinical outcomes; these questions require prospective evaluation in real-world laboratory and clinical workflows.

The relationship between TG concentration and actual HGB interference provides a further explanation for why a single biochemical measure cannot fully capture lipemic effects. Although TG and Bias_PR were strongly correlated (rho=0.734), the association was not deterministic. Lipemic interference in photometric HGB measurement depends primarily on plasma turbidity, which is influenced not only by triglyceride concentration but also by lipoprotein particle size, composition, and distribution; previous studies have demonstrated imperfect correspondence between TG concentration and lipemic or turbidity indices (1, 5, 6). Consistent with this concept, adding log2(TG) and TC produced a statistically detectable but clinically modest gain, reducing HGB MAE from 1.66 to 1.48 g/L, an approximately 11% relative reduction, while the absolute improvement was only 0.18 g/L. From a practical laboratory perspective, this limited incremental benefit suggests that routine CBC parameters already capture much of the predictive information relevant to lipemia-associated HGB distortion, whereas biochemical lipid measurements provide only modest complementary information. Accordingly, requiring simultaneous TG/TC measurements solely for model application may not be justified, particularly when the intended use is rapid automated decision support based on data already available from the CBC analyzer.

Mathematical coupling is an important methodological consideration because HGB-I is included in the definition of Bias_PR and MCH and MCHC are themselves related mathematically to HGB and other erythrocyte measurements. The sensitivity analyses showed that, even after HGB-I, MCH-I, and MCHC-I were excluded from Bias_PR prediction, average HGB MAE remained close to that of the primary model (1.71 vs. 1.66 g/L). This finding suggests that the observed predictive performance was not solely dependent on the most direct algebraic relationships, but it should not be interpreted as definitive evidence of fully independent predictive information. The strict anti-coupling model also showed lower explained variance, greater RMSE, and poorer performance among specimens with Bias_PR >10%, indicating that HGB-related indices contributed materially to prediction, particularly in the high-bias tail. Mathematical coupling should therefore be regarded as a transparent methodological limitation and sensitivity-analysis issue rather than interpreted in causal terms.

The error structure identifies where further model development is most needed. Importantly, residuals became increasingly negative at higher OOF-predicted Bias_PR values, while the largest HGB errors were concentrated in the severe and extreme observed Bias_PR groups. This pattern is compatible with regression toward the center when extreme observations are relatively sparse. Accordingly, favorable overall R^2^ or MAE values should not be interpreted as uniform accuracy across the full interference range. For laboratory application, severe or extreme lipemic specimens may require confirmatory direct correction rather than exclusive reliance on a model-derived HGB estimate. Future development should prioritize enrichment of severe and extreme lipemic specimens, prospective assessment of tail performance, and potentially uncertainty-aware outputs or prediction intervals.

Several features strengthen this study. The model was developed from real clinical lipemic specimens rather than solely from artificial lipid-emulsion experiments; all 283 included specimens were from unique patients and had a specimen-level plasma-replacement comparator that met the prespecified RBC quality-control criterion; and all principal performance estimates were based on nested OOF predictions that separated hyperparameter optimization from performance evaluation. The study also evaluated clinically relevant comparators, patient-level paired errors, agreement, residual behavior, model interpretation, mathematical-coupling sensitivity, and extreme errors. These design choices are consistent with contemporary recommendations for transparent prediction-model development and evaluation (15–17).

Several limitations should also be recognized. First, this was a single-center study conducted on a single hematology analyzer platform (Sysmex XN-9000). The quantitative relationships among HGB-I, HCT-I, MCH-I, MCHC-I, and lipemia-associated bias may depend on analyzer-specific measurement principles, reagent systems, instrument calibration, and laboratory workflow. The present model should therefore be regarded as platform- and setting-specific, and its predictive performance should not be assumed to transfer directly to other hematology analyzers or laboratories. External validation across independent centers, different Sysmex XN-series configurations, and other analyzer platforms will be necessary to assess transportability; where systematic platform differences are present, recalibration or platform-specific retraining may be required before implementation. Second, plasma replacement is a clinically practical comparator but is not an absolute reference measurement procedure and remains susceptible to procedural variation. Although 27 candidate specimens that failed the prespecified RBC consistency criterion were excluded, this may reduce representation of technically difficult cases and underscores the need to report cohort flow transparently. Third, this study provides internal validation only. Nested cross-validation reduces optimism in model-development estimates but cannot substitute for independent temporal or multicenter validation. Fourth, the model was developed among specimens already identified as lipemic and should currently be regarded as a conditional bias-estimation model for suspicious or lipemic specimens rather than a universal screening algorithm for all CBC samples. Fifth, relatively few specimens occupied the most extreme portion of the Bias_PR distribution, where prediction errors were greatest. Finally, this study evaluated analytical agreement rather than downstream clinical consequences such as changes in anemia classification, transfusion decisions, patient management, or clinical outcomes.

## 5 Conclusions

Routine CBC parameters can enable continuous estimation of lipemia-associated HGB bias and may support automated identification of specimens requiring reflex testing or analytical correction. The model is not intended to replace established correction procedures, and independent multicenter prospective validation is required before clinical implementation.

## Data Availability

The original contributions presented in the study are included in the article/Supplementary Material, further inquiries can be directed to the corresponding author.

## Supplementary material

Supplementary Figure S1 Correlation, collinearity, and arithmetic-consistency analyses for the five primary CBC predictors. (A) Pearson correlation matrix. (B) Variance inflation factors, with the reference value of 5 indicated by the dashed line. (C) Reported MCHC-I versus MCHC recalculated from HGB-I and HCT-I; the dashed line denotes identity and the largest discrepancies are highlighted.

Supplementary Figure S2 Triglyceride association and TG-Bias_PR discordance. (A) Relationship between triglyceride concentration and observed Bias_PR on a logarithmic TG scale; the fitted curve summarizes the nonlinear trend. (B) Difference between observed Bias_PR and the value expected from the TG-only relationship. Dashed horizontal lines indicate ±5 percentage points; specimens outside these limits are classified as lower or higher than expected, and selected extreme cases are labeled.

Supplementary Figure S3 Large-error analysis for individualized HGB_ML. (A) Absolute HGB errors for all 283 specimens ranked from largest to smallest; the dashed line marks 5 g/L, and the 16 specimens above this threshold are highlighted. (B) Observed versus OOF-predicted Bias_PR for these 16 specimens, colored by observed Bias_PR severity; the dashed diagonal line denotes perfect agreement and case identifiers are shown.

Supplementary Figure S4 Secondary classification analysis for Bias_PR >4%. (A) ROC curves for OOF-predicted Bias_PR and MCHC-I, with AUCs of 0.963 and 0.972, respectively. (B) Confusion matrix for the prespecified predicted Bias_PR >4% rule: 208 true negatives, 5 false positives, 6 false negatives, and 64 true positives. Sensitivity was 91.4%, specificity 97.7%, positive predictive value 92.8%, negative predictive value 97.2%, and accuracy 96.1%. This analysis was secondary to continuous Bias_PR prediction and individualized HGB correction.
